# Impacts of the Goldmining and Chronic Methylmercury Exposure on the Good-Living and Mental Health of Munduruku Native Communities in the Amazon Basin

**DOI:** 10.3390/ijerph18178994

**Published:** 2021-08-26

**Authors:** Rafaela Waddington Achatz, Ana Claudia Santiago de Vasconcellos, Lucia Pereira, Paulo Victor de Sousa Viana, Paulo Cesar Basta

**Affiliations:** 1 Programa de Pós-Graduação em Psicologia Clínica do Instituto de Psicologia, Universidade de São Paulo (USP), Av. Professor Mello Moraes, 1721-Butantã, São Paulo 05508-030, Brazil; rafa.achatz@gmail.com; 2 Laboratório de Educação Profissional em Vigilância em Saúde, Escola Politécnica de Saúde Joaquim Venâncio, Fundação Oswaldo Cruz (EPSJV/Fiocruz), Av. Brasil, 4365-Manguinhos, Rio de Janeiro 21040-900, Brazil; anacsvasconcellos@gmail.com; 3 Programa de Pós-Graduação em Antropologia, Universidade Federal da Grande Dourados (UFGD), Rodovia Dourados, Km 12, Unidade II, 364, Itahum, Dourados 79804-970, Brazil; luciakaiova@gmail.com; 4 Centro de Referência Professor Hélio Fraga, Escola Nacional de Saúde Pública, Fundação Oswaldo Cruz (CRPHF/ENSP/Fiocruz), Estrada de Curicica, 2000, Curicica, Rio de Janeiro 22780-195, Brazil; paulovictorsviana@gmail.com; 5 Departamento de Endemias Samuel Pessoa, Escola Nacional de Saúde Pública, Fundação Oswaldo Cruz (ENSP/Fiocruz), Rua Leopoldo Bulhões, 1480, Manguinhos, Rio de Janeiro 21041-210, Brazil

**Keywords:** illegal mining activities, methylmercury exposure, Good-Living, mental health, Munduruku, Amerindian people, Brazilian amazon, environmental pollution, cosmopolitics

## Abstract

This paper is an exploratory study that examines the illegal goldmining impacts on Munduruku communities’ “Good-Living” (*Xipan Jewewekukap*) and explores the possible relationship between chronic methylmercury (MeHg) exposure and the worsening mental health conditions in three villages in the Middle-Tapajós River, Brazilian Amazon. The region has been experiencing a long-lasting threat of goldminers’ invasions. A total of 109 people were interviewed and evaluated. Total mercury (THg) exposure levels were evaluated through hair samples analysis, from which MeHg exposure levels were calculated. The Geriatric Depression Scale—Short Form (GDS-SF) was used as a screening tool in order to assess mental health indicators. Brief non-structured interviews were carried out to investigate how goldmining is impacting the communities Good-Living. A Poisson regression model was used to estimate the possible association between mental health indicators (assessed through the GDS-SF) and the following independent variables: (i) mercury exposure level (<10.0 μg/g vs. ≥10.0 μg/g), (ii) self-reported nervousness, (iii) self-reported irritability, (iv) age group, and (v) monthly income. The analysis revealed high levels of mercury in hair samples (median: 7.4 µg/g, range 2.0–22.8; 70% and 28% of the participants had THg levels ≥6.0 and ≥10.0 µg/g, respectively) and pointed to a tendency in which higher levels of methylmercury exposure (Hg ≥ 10.0 µg/g) could be linked to worse mental health indicators. Although the GDS-SF has presented limitations due to the Munduruku sociocultural context, our findings suggest a tendency of worse mental health indicators in participants presenting high levels of MeHg exposure. Despite this limitation, the qualitative approach indicates an evident association between the impacts of goldmining and the Munduruku people’s decreasing autonomy to maintain a Good-Living on their own terms, pointing to the importance of carrying out new investigations, especially considering longitudinal studies with qualitative methodologies and ethnographic approaches.

## 1. Introduction

The Munduruku are Amerindian people who are part of the Munduruku linguistic family and integrate the Tupi linguistic trunk. They are traditionally located at the Tapajós Valley, in Brazil. Currently, the majority live on the banks of navigable rivers, in savanna regions of the Amazon rainforest located in the states of Pará, Amazonas, and Mato Grosso [1,2]. Presently, the Munduruku population is estimated at approximately 12,000 people distributed in more than 120 villages. The contemporary battles fought by the Munduruku are still focused on guaranteeing the integrity of their territory, permanently threatened by pressures not only from illegal goldmining activities, land grabbing, and logging but also from large government projects that include the expansion of agribusiness over traditional territories as well as the construction of hydroelectric dams and a large waterway in the Tapajós River. Furthermore, like other native people in Brazil, the Munduruku people have also been striving against colonial and environmental racism [3,4].

Since the 19th century, Munduruku people have been struggling to protect their territory from the invasion of the economic and missionary expansion fronts. Between 1880 and 1920, the rubber exploitation and the religious missions’ proselytism played an important role in the Munduruku territory’s invasion. In the late 1950s, after the fall of rubber prices on the international market, a gold rush took place in the Tapajós valley. The gold rush was intensified during the civil–military dictatorship, mostly after 1972 (when the *Transamazônica* highway began its construction) and had its peak in the period between 1975 and 1990. Highly profitable for non-Amerindian people, the exploitation of gold left a noxious legacy for the local population, not only related to the harm it caused to the environment and its inhabitants (such as uncontrolled deforestation, major excavations, and contamination of waters by mercury) [5,6,7,8,9] but also due to the psychosocial impacts that, since then, have been contributing to making Munduruku communities vulnerable.

At the beginning of the 21st century, with the developmentalist policies of the Brazilian Workers Party’s governments and the impacts of the global economic crisis in Brazil, there was a further expansion in the search for gold in the Tapajós region, which was intensified by the current extreme right-wing Brazilian government agenda. Illegal small-scale goldmining (ASGM) has significantly expanded in the Amazon in recent decades [10], being an important cause of deforestation and environmental degradation [11,12,13]. The mercurial form used in artisanal and small-scale mining is metallic mercury, also known as elemental mercury (Hg0). During the extraction process, a large part of the mercury used is released into rivers and undergoes a methylation process (mediated by aquatic microorganisms), producing methylmercury, which is extremely harmful to human health and the ecosystem.

Much of the danger attributed to methylmercury (MeHg) refers to its capacity for bioaccumulation and biomagnification in aquatic food chains and due to its highly neurotoxic potential. Since it is highly fat-soluble, it can cross the blood–brain barrier and reach the central nervous system. Most traditional peoples of the Amazon are large consumers of fish, and in many cases, it represents the main or the only source of animal protein in their diet [14,15]. As such, the presence of goldmines in traditional territories converts the consumption of fish, which was once considered to be healthy, into a risky behavior, since the mercury used in the process contaminates the rivers and is incorporated into the food chain, contaminating humans [16,17,18].

In the literature, mercury exposure is linked to the manifestation of psychological symptoms, which differ in extent and severity according to multiple etiological factors, such as the mercurial chemical form (organic or inorganic), the duration and levels of exposure (acute or chronic), and the pathway of contamination. Most of the studies on the relationship between mercury exposure and the manifestation of psychological symptomatology report on cases of acute contamination by methylmercury or occupational exposure to inorganic mercury. Among other health problems, acute MeHg exposure has been related to the manifestation of psychological symptoms such as depression, anxiety, insomnia, irritability, and decreased attention and memory [19,20]. Some studies suggest that chronic MeHg exposure can produce neuropsychological impacts, affecting cognition, memory, IQ, verbal fluency, and psychomotor performance [21,22,23]. However, there is still little research focusing specifically on the psychological effects of chronic exposure to methylmercury through diet [24].

Be that as it may, the impacts of goldmining on the Munduruku people’s mental health indicators go far beyond chronic exposure to methylmercury, since it undermines their possibilities to maintain a *Xipan Jewewekukap*, a Munduruku concept that could be translated as “Good-Living”. Good-Living (“*Bem Viver*” in Portuguese) is a translation of Amerindian concepts such as *Teko Porã* (in Guarani-Mbya), *Suma Qamaña* (in Aymara), and *Sumak Kawsay* (in Quechua), only to mention some examples. The definition of what is a Good-Living is different for each Amerindian people and may vary from generation to generation. However, there are some common points between those concepts translated as Good-Living: they belong to philosophical systems that propose an ecological conception of reality and consider that nature is a living being (and not an object or a source of resources). Thus, these philosophical systems recognize that the lives of all beings that inhabit the Earth (including human beings) are deeply intertwined. In the last decades, the possibilities of non-capitalistic definitions of what may be Good-Living have been an important topic of debate for Latin American social movements, public health agents, psychologists, and social scientists [25,26,27,28].

Many Munduruku leaders have been tireless in warning about how the goldmining is radically damaging their *Xipan Jewewekukap*. In an interview given in 2016, Maria Leusa Munduruku, an important leader in the fight to protect Munduruku territory, said [29]:

*For us, the river is the place where we take our food from. We drink the river, we bathe in the river. For us the breast milk, as we say, is the riverbed. The same river also runs in people’s veins. Without the river, there will be no “us”. Without water, nobody can live. Our river is our mother. So it is with the forest. They are sacred because they came from a story in which our ancestors made the Tapajós River with water squeezed from *tucumã *woodworms.*

In 2016, the Pariri Amerindian Association (representing the Munduruku people living in the Middle-Tapajós Region) sent a letter to the Oswaldo Cruz Foundation to request an assessment of mercury contamination in Amerindian people, given the increasing presence of goldminers in the area. This work is an exploratory study that integrates a broader effort to follow this request and investigate the impacts of goldmining in the Amazon on human health and the environment, focusing on the exposure to methylmercury (for more details, see Basta et al. [30]).

With this requesting in mind, the purpose of the present paper is (i) to examine the impacts of illegal goldmining on the Munduruku Amerindian communities’ Good-Living and (ii) to explore the possible relationship between MeHg exposure and the worsening mental health conditions reported by the residents living in the studied communities. The following analysis is based on data collected through home visits and interviews with the participating families during fieldwork at Munduruku communities.

## 2. Materials and Methods

This study integrates quantitative and qualitative approaches, the latter being predominantly ethnographic. We consider that the qualitative approach enables (i) a more symmetrical dialogue with Munduruku knowledge regimes and (ii) the research team to ask questions and pay attention to variables that escape what was foreseen in the initial methodological design. In other words, it enables accessing variables that cannot be evaluated using psychometric or psychiatric scales, which nonetheless are fundamental variables to be considered in the study.

### 2.1. Sampling

A cross-sectional study was carried out seeking to investigate mental health indicators in Munduruku people over 12 years of age living in the *Sawré Muybu*, *Poxo Muybu*, and *Sawré Aboy* Munduruku villages, which are located in the Middle-Tapajós region. Those villages were included in the sample following the request of the *Pariri* Indigenous Association, which represents the Munduruku People living in the Middle-Tapajós River. Those communities are located in the *Sawré Muybu* Indigenous Land (IL) and are chronically exposed to methylmercury as a result of the increasing illegal goldmining activity in the region. The *Sawré Muybu* IL is located in the Southwest of Pará State, in an area of 173,178 hectares situated between the municipalities of Itaituba and Trairão. The demarcation process of *Sawré Muybu* IL, which is traditionally occupied by the Munduruku people, started in 2007 and remains uncompleted. For the Munduruku people, the territory where the *Sawré Muybu* IL is located is extremely important since it is known as *Daje Kapap Eipi*—the place where the Tapajós River emerged according to Munduruku cosmogony.

A population census was carried out in those communities, and all residents aged 12 years or over were invited to participate in the study. Therefore, probabilistic sampling methods were not used to select participants. In total, 109 people were included in the study. Two residents of the *Sawré Muybu* community were excluded: a 73-year-old woman who had speech and mobility difficulties due to a stroke and a 15-year-old boy who had congenital chronic non-progressive encephalopathy due to perinatal complications and neurological symptoms resulting from cerebral palsy.

### 2.2. Fieldwork

The fieldwork was held over 11 days between October and November 2019. The data that support the following analysis were obtained through home visits, interviews with the participating families, and the collection of hair samples, used as a biomarker of total mercury (THg) exposure. Four researchers participated in home visits and interviews with families: an Amerindian anthropologist (L.P.), a biologist (A.C.S.V.), a nurse (P.V.S.V.), and a psychologist (R.W.A.). The interviews were conducted based on a questionnaire specially prepared for this research, based on previous experiences of our research group [30].

### 2.3. Data Collection Instruments

The data collection instrument was a questionnaire structured in thematic modules, through which we sought to (a) characterize the physical and demographic structure of the visited households; (b) characterize the health situation in the family and in the community, searching for psychological self-reported symptoms; and (c) characterize the dietary pattern of families, with emphasis on the fish consumption. Concerning mental health indicators, the Geriatric Depression Scale—Short Form (GDS-SF) was used to screen depressive symptoms. Brief non-structured interviews were carried out to investigate how the participants perceived the goldmining impacts on their right to self-determinatively maintain a Good-Living.

The meaning of each question was carefully explained to participants and, if necessary, translated into Munduruku. The questionnaire was applied with the help of Munduruku health agents who work in the communities and/or with the help of village leaders, including chiefs (*caciques*) and teachers. Answers were recorded on electronic forms with the help of portable electronic devices (tablets), and there was no use of paper forms.

#### 2.3.1. Geriatric Depression Scale—Short Form

**Figure 1:**
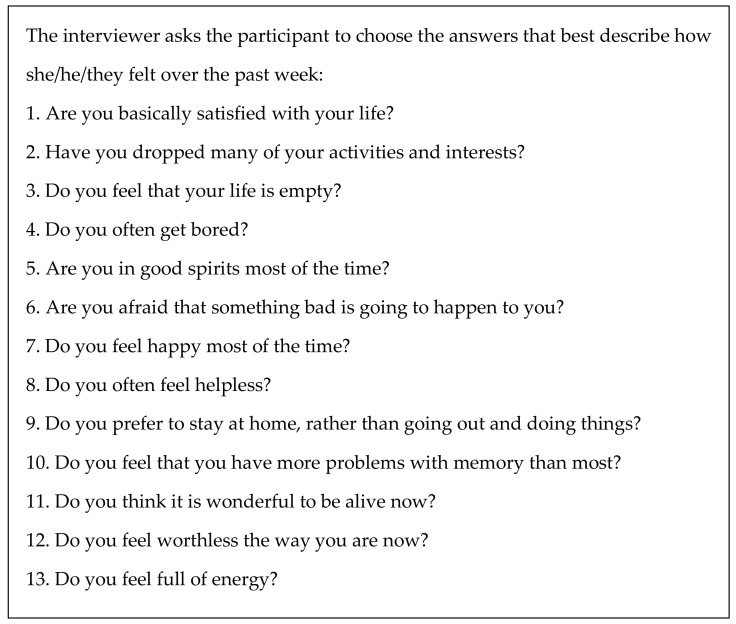
Geriatric Depression Scale—Short Form (Sheikh J.I., Yesavage J.A., 1986).

The GDS-SF (Figure 1) is a reduced version of the original scale (which is composed of 30 questions) and aims to screen depressive symptoms [31,32]. The scale ranges from zero (absence of depressive symptoms) to fifteen points (maximum score for depressive symptoms).

Even though the GDS-SF is designed to evaluate depressive symptoms in the elderly, it can be applied to a younger population for the following reasons: it is composed of easily understood questions, it has small variation in answer possibilities (yes/no), it can be applied by any trained interviewer, and it requires little time for application. Additionally, research indicates that the GDS-SF shows good diagnostic sensitivity and specificity for young and middle-aged adults [33]. It is worth remembering that the GDS-SF has widespread use in clinical practice and is commonly used to assess young adults in Brazil, especially those with little access to formal education.

According to Abas M. et al. [34] experience and considering culture-specific criteria, a cutoff score ≥4 was employed to screen the presence of depressive symptoms. It is important to emphasize that for the present analysis, this cutoff score was employed only to access mental health indicators and not to diagnose any kind of mental disorder.

#### 2.3.2. Brief Non-Structured Interviews

Brief non-structured interviews were performed to access the goldmining impacts on the Good-Living of the communities. The interviewers asked the participants how they had been feeling in the last months. If the person answered that she/he had not been feeling well, the interviewer asked if she/he would like to specify how they had been feeling and which were their complaints about it. If necessary, the interviewer gave examples of uncomfortable emotional states (such as nervousness, irritability, sadness, anxiety, fear, grief, and outbreaks of rage), which were usually translated into the Munduruku language. Some of these interviews triggered testimonies from the participants, who detailed how they understood the impacts of goldmining in their territory. These extended interviews were fundamental to access how the impacts of goldmining on Munduruku people’s Good-Living go far beyond the impacts of methylmercury contamination.

Hearing Munduruku leaders’ testimonials is a fundamental step in order to better understand the scale of the noxious impacts promoted by goldmining in their territory. These impacts go beyond the impacts on mental health, affecting the socio-cosmological organization of the Munduruku people. We assume that Amerindian knowledge systems are different and no less legitimate than Western knowledge systems [35], and we consider that it is necessary to acknowledge ontoepistemological legitimacy to non-Western people [36,37]. As such, in order to take Munduruku people seriously and to reduce the misunderstandings that are inherent to cultural translation [38], it is necessary to listen carefully to their analyses and experiences about how goldmining and methylmercury exposure affect them.

#### 2.3.3. Mercury Exposure Biomarker

Hair samples were collected from the occipital region of all participants using stainless steel dissection scissors. The samples were packed in paper envelopes and individually identified. Analyses of THg levels in the participants’ hair samples were performed at the Environment Section of the Evandro Chagas Institute of the Ministry of Health, located in Belém-PA. The health risk assessment was carried out according to the methodology proposed by WHO [39]. For more details on the analysis of hair samples and the discussion about safe levels of methylmercury exposure standards, see Basta et al. [30].

There is no consensus on the thresholds of lower levels of mercury in hair samples that may impact the onset of psychological effects. The World Health Organization and some studies carried out in the Amazon region [40,41,42] propose a level of mercury in hair samples ≥6.0 µg/g as an indicator of general health risk. Concerning specifically neurological abnormalities associated with the consumption of methylmercury contaminated fish, some studies propose a level of mercury in hair samples ≥10.0 µg/g as an indicator of neurological alteration risk. Research performed in the Amazon since the 1990s has identified adult individuals with neurological alterations associated with THg levels in hair samples varying between 10 and 20 μg/g [43,44,45,46]. According to Oliveira et al. [47], Munduruku indigenous adults with Hg exposure level ≥10µg/g presented twice as high chances of cognitive development deficits (Prevalence Ratio-PR: 2.2; CI 95%:1.13–4.26) in Brief Cognitive Screening Battery as well as in the verbal fluency test (PR: 2.0; CI 95%:1.18–3.35). Similar findings were reported in riparian populations in Bolivia and Suriname [48,49].

### 2.4. Statistical Analysis

A descriptive analysis of the participants was conducted according to clinical and sociodemographic variables of interest, including gender (female/male), age group (12–19; 20–29, or ≥30 years old), schooling (0 to 4 years, 5 to 9 years, or >9 years of formal education), marital status (married or single/widow), village of residence (*Sawré Muybu*, *Poxo Muybu*, or *Sawré Aboy*), regular monthly income (yes/no), psychological self-reported symptoms, such as nervousness (yes/no), irritability (yes/no), besides school failure (yes/no), physical activity restriction (yes/no), previous treatment for Malaria (yes/no), previous hospitalization (yes/no), and MeHg exposure (<10.0 µg/g vs. ≥10.0 µg/g). Moreover, the mental health indicators (assessed through the GDS-SF <4 vs. ≥4) were compared according to the abovementioned variables.

To estimate the prevalence of exposure, the proportion of people over 12 years of age who had mercury levels ≥10.0 µg/g in the sampled population in the study region was considered. The prevalence was presented for the three studied villages: *Sawré Muybu*, *Poxo Muybu*, and *Sawré Aboy*.

In order to contrast clinical and sociodemographic variables with mental health indicators (assessed through the GDS-SF <4 vs. ≥4), we used Pearson’s Chi-squared test or Fisher’s exact test. We also used the Kruskal–Wallis test to evaluate the differences in Hg levels between villages.

A Poisson regression model was used to estimate the possible association between mental health indicators (assessed through the GDS-SF <4 vs. ≥4) and the following independent variables: (i) mercury exposure level (<10.0 μg/g vs. ≥10.0 μg/g), (ii) self-reported nervousness, (iii) self-reported irritability, (iv) age group, and (v) monthly income. Prevalence ratio (PR), with the respective confidence interval of 95%, was used as an association measure.

Variables with *p*-value < 0.10 in the simple analysis were selected and included in the model. Variables that presented significance levels of 5% (*p*-value < 0.05) remained in the final model. Age group and income also remained in the final model in order to control the effects of the sociodemographic variables on the mental health indicators.

The data were analyzed using SPSS (Statistical Package for the Social Sciences), version 9.0 (Chicago, IL, USA).

## 3. Results

During fieldwork, 109 people over 12 years old provided hair samples and answered the mental health questionnaires: 46 from the *Sawré Muybu* village, 39 from *Poxo Muybu* and 24 from *Sawré Aboy* (Table 1). A total of 35 households were visited in the *Sawré Muybu* IL, 20 in *Sawré Muybu* village, 8 in *Poxo Muybu* village, and 7 in *Sawré Aboy* village. Most families reported having regular incomes from social benefits and or salaries. A total of 53 women (48.6%) and 56 men (51.4%) participated in the study. The age of participants ranged from 12 to 72 years old (mean: 27.4 years old; standard deviation: 13.8 years old).

The distribution of participants according to age group was heterogeneous among the studied villages. There was a predominance of people aged from 12 to 19 years old in *Sawré Aboy* and *Poxo Muybu* villages, with the presence of 12 (50%) and 18 (46%) participants, respectively, in contrast to only 7 (15%) in the *Sawré Muybu* village. On the other hand, the *Sawré Muybu* village had the highest concentration of people aged between 20 and 29 years old, with the presence of 25 participants (54%), in relation to only 5 (21%) and 8 (21%) in the *Sawré Aboy* and *Poxo Muybu* villages, respectively. There was a similar concentration of people aged 30 years or more in the three villages: 7 (29%) in *Sawré Aboy*, 13 (33%) in *Poxo Muybu,* and 14 (30%) in *Sawré Muybu*.

### 3.1. Mercury Exposure

The analysis of mercury levels for the 109 participants who provided hair samples revealed that the average concentration level was 8.4 (±4.2) µg/g and the median was 7.4 µg/g, ranging between 2.0 and 22.8 µg/g. In general, the prevalence of exposure reported, considering THg hair levels ≥10.0 µg/g, was 28%.

The prevalence of methylmercury exposure, considering THg hair levels ≥10.0 µg/g, was uneven among the investigated communities (*p*-value = 0.001). In the *Poxo Muybu* village, 13.3% (*n* = 4) of the sampled residents had THg hair levels ≥10.0 µg/g, while in *Sawré Muybu* and *Sawré Aboy* villages, respectively, 30% (*n* = 9) and 57% (*n* = 17) of the residents had THg hair levels ≥10.0 µg/g. In other words, the prevalence of methylmercury exposure considering the limit of 10.0 µg/g was 2.3 times higher in *Sawré Muybu* and 4.3 times higher in *Sawré Aboy* when compared to the exposure rates observed in *Poxo Muybu*.

### 3.2. GDS-SF

Out of the 109 participants who answered the GDS-SF, 32% scored zero or one points, 34% scored two or three points, 17% scored four or five points, 12% scored six or seven points, and 6% scored between eight and ten points. Even though the scale reached up to 15 points, no participant obtained a score higher than 10 points. Therefore, 35% of the participants (*n* = 37) scored ≥4 points.

When considering the scores obtained in each item by the 109 participants as a whole, the lowest scores refer to Items 11 (since only 2% of the participants answered “no” to the question “Do you think it is wonderful to be alive now?”) and 1 (since only 6% of the participants answered “no” to the question “Are you basically satisfied with your life?”), and the highest scores refer to Items 6 (since 34% of the participants answered “yes” to the question “Are you afraid that something bad is going to happen to you?”) and 14 (since 32% of the participants answered “yes” to the question “Do you feel that your situation is hopeless?”).

The prevalence of scores ≥4 did not vary significantly according to gender (*p*-value = 0.416), schooling (*p*-value = 0.339), income (*p*-value = 0.540), marital status (*p*-value = 0.597), or age group (*p*-value = 0.376) (Table 1). Nevertheless, it is noteworthy that only participants aged between 20 and 25 years old scored 9 or 10 points and only participants aged between 20 and 35 years old scored ≥8 points (Table 2).

In the multivariate analysis (PR adjusted), the presence of depressive symptoms (indicated by the score ≥4 on the GDS-SF) appears to be associated with THg hair levels ≥ 10 µg/g (PR = 1.8; CI 95%: 1.1–3.0), complaints of irritability (PR = 3.0; CI 95%:1.9–4.9), and nervousness (PR = 2.1; CI 95%: 1.3–3.3), even when controlling the income effect (PR = 1.1; CI 95%: 0.6–1.9) and age group effect (PR = 0.7; CI 95%: 0.4–1.5; (PR = 1.4; CI 95%: 0.8–2.5) (Table 3).

In other words, people with THg levels ≥10 µg/g are 1.8 times more likely to manifest depressive symptoms according to the GDS ≥ 4, when compared to people with THg levels < 10 µg/g. Participants who reported feeling excessive irritability are 3.0 times more likely to manifest depressive symptoms according to the GDS ≥ 4, in contrast to participants who did not complain of irritability. In addition, people who complained of excessive nervousness are 2.1 times more likely to manifest depressive symptoms according to the GDS ≥ 4, when compared to people who did not report feeling nervous, even when controlling the effect of the income and the age group.

### 3.3. Brief Non-Structured Interviews

Besides the individual mental health indicators assessed through the GDS-SF, the non-structured interviews triggered testimonials about how goldmining is affecting these Munduruku villages collectively.

The conversations held with Munduruku people during fieldwork suggest that there is a close relationship between the impacts of illegal goldmining and Munduruku people’s decreasing Good-Living. This is mainly related to the fact that goldmining activities in Munduruku territory undermine the Munduruku people’s possibility to maintain Good-Living on their own terms. The Munduruku elders and leaders explained that, in order to cultivate the Good-Living of each community and each person, it is necessary to take care of the relationships between the different inhabitants of the forest. As one of the villages’ leaders said:

*The forest (*auadip*) is beautiful to me; it makes me joyful and makes me recognize who I am. If I am not in the forest, I feel strange, unfocused, and shattered. And if the forest is being assaulted and hurt, I get hurt. I get sick if the forest gets sick, because my body and my speech are also made of the forest.*

## 4. Discussion

### 4.1. Methylmercury Exposure and Mental Health Impacts

The relationship between mercury exposure and psychological disorders is determined by multiple etiological factors, such as the mercurial form (organic or inorganic), the duration and levels of exposure, and the pathway of contamination [50,51].

Cases of acute inorganic mercury contamination have been associated with symptoms such as hallucinations, delusions, insomnia, suicidal tendencies, loss of memory, and manic-depressive behavior [52]. Chronic inorganic mercury contamination is usually due to occupational exposure to the metal and has been related to anxiety, irritability, depression, memory problems, and erethism (popularly known as “mad hatter disease”) [53,54,55]. Cases of chronic inorganic mercury exposure were also discussed by studies on the mental health of individuals with mercury dental amalgam, which suggested that long-term exposure to small amounts of inorganic mercury can produce devastating effects after several years without the onset of symptoms [56].

Cases of acute organic mercury exposure were also related to the manifestation of depressive symptoms. Several outbreaks of methylmercury contamination have been reported around the world since 1956, when an outbreak of acute methylmercury contamination took place in Minamata Bay, Japan [57]. Another well-known episode took place in Iraq in the 1970s when thousands of people were hospitalized due to the consumption of grains treated with methylmercury or ethylmercury fungicides [58]. Mayhazati [59] reported on the psychological effects of acute methylmercury contamination in patients in Iraq whose total blood levels of mercury ranged between 320 and 4260 ng/mL, with an average of 2118 ng/mL. Of the 43 patients studied, 32 (74.4%) showed depressive symptoms and 19 (44.2%) manifested irritability. The average blood levels of mercury (both organic and inorganic) were considerably higher in depressed patients than in non-depressed patients. Nevertheless, the author highlights that mercury poisoning may be one of the various contributory factors in causing depression in the patients, who were also suffering physically and financially and handling the stress and grief produced by the disruption of their lives.

However, there is still little research on the impacts of methylmercury exposure in cases of chronic toxicity, especially considering its psychological impacts. Most of the studies focus on the neuropsychological impacts of chronic MeHg exposure, such as cognition, memory, sleep, and psychomotor performance [21,22,23]. It is not consensual that chronic exposition to methylmercury by diet has direct impacts on mental health indicators. Junior et al. [20] analyzed emotional and motor symptoms of riverside dwellers exposed to methylmercury by diet in the municipalities of Itaituba and Acará, in Pará, Brazil. The mean levels of THg in Itaituba (9.15 μg/g) were significantly higher than in Acará (0.67 μg/g), but emotional symptoms were identified in 26 (26.5%) participants from Itaituba and in 24 (52.2%) from Acará, suggesting that the exposure to Hg may not be the causal factor of these emotional symptoms.

Tracing the relationship between MeHg chronic exposure and psychological symptomatology is difficult for some reasons, for example: (1) Chronic methylmercury exposure may be a silent pandemic. It is considered by some authors as a silent damage [60]—unlike cases of acute contamination, chronic exposure to lower doses of methylmercury usually produces subclinical manifestations, which are insidiously and cumulatively manifested over a long period of time; (2) Multiple factors contribute to the manifestation of psychological symptoms. Mental health indicators are never determined by a single factor but are produced by a multifactorial combination. For instance, besides methylmercury exposure, goldmining in the Tapajós river produces several other negative impacts on the communities’ mental health and Good-Living, as it undermines environmental relations that are imperative to provide the conditions for Good-Living on the terms of the Munduruku people and other native people from the Amazon.

### 4.2. Methylmercury Exposure and Mental Health Indicators

Data suggest that the variation on the scores obtained in the GDS-SF according to the levels of THg concentration in hair samples is statistically significant. Even though the sampled population is predominantly young, these results may point to a tendency in which higher levels of methylmercury exposure could be linked to worse mental health indicators. The highest scores are concentrated in *Sawré Aboy* village, where there is the highest prevalence of MeHg exposure, and in *Sawré Muybu*, where the population is older. Additionally, the prevalence of participants who scored ≥4 points in the GDS-SF is higher in participants who had mercury levels above 10.0 µg/g, in comparison to participants who had mercury levels under 10.0 µg/g—according to GDS-SF scores, people with THg ≥ 10 µg/g are 1.8 times more likely to manifest depressive symptomatology, when compared to people with THg levels < 10 µg/g.

The variations considering the overall points scored in each item of the GDS-SF are also relevant. Most of the participants answered that they felt happy and that they were satisfied with their lives. Nevertheless, many participants also answered in the GDS-SF that they felt hopeless and that they were afraid that something bad could happen to them or their relatives. Such data may suggest that the greatest sources of anguish and anxiety are related to future prospects. Given the situation of the progressive invasion and destruction of the Munduruku territories, the future presents itself as full of uncertainties and daunting prospects.

It is noteworthy that such data can only point to tendencies since the instruments of data collection should be better adapted to Munduruku sociocultural specificities. Moreover, it is possible that psychological symptoms are not manifesting since these are cases of chronic contamination by methylmercury that could be described as a “silent pandemic”. Considering that the entire population is exposed to considerable levels of mercury, it is difficult to establish relationships between the prevalence of methylmercury exposure and the results of the scales and reported symptoms. The goldmining itself has devastating impacts on Munduruku people’s mental health conditions, whether mercury exposure is ≥10.0 µg/g or not. Therefore, MeHg exposure may be increasing the negative impacts of goldmining activities on the Good-Living of the Munduruku people and other inhabitants of the forest.

### 4.3. Limitations

The limitations of this study are mostly related to the data collection instruments employed, the limited size of the sample considering the Munduruku population, and the context of the application of the questionnaires. Such limitations point to the importance of carrying out new investigations, especially considering longitudinal studies with qualitative methodologies articulated to ethnographic research.

Since the interviews were conducted at the participants’ homes, in most cases, the entire family was present during the interview. The lack of privacy, combined with the lack of intimacy between interviewer and interviewee, may have contributed to people being embarrassed to answer about issues that could be sensitive. Additionally, some questions can be perceived as invasive to participants if asked without well-established trust bonds.

Linguistic and cultural translation was also a challenge in conducting the interviews. Like other Amerindian people, Munduruku knowledge and medical practices are based on onto-epistemological assumptions that differ from those postulated by biomedical or psychological knowledge. As such, it is inaccurate to translate Munduruku nosological categories into categories like “depressive symptomatology”, “psychological disorder”, or “mental health” [61]. Therefore, the instruments utilized in this research may produce equivocal comprehension [38] regarding Munduruku ontological and sociocultural specificities, since the GDS-SF is not validated for Munduruku people.

There is no consensus on the reliability of the GDS-SF in different cultural contexts. Some studies support that the GDS-SF is reliable for the cultural contexts investigated, despite cultural differences [62,63]. Some authors argue that the GDS-SF cutoff score may vary according to ethnocultural specificities. Almeida and Almeida [64] propose a cutoff score ≥5 to determine the presence of depressive symptoms in elderly Brazilian people. In turn, Abas M. et al. [34] recommend a cutoff score of ≥4 to detect significant forms of depression in older African–Caribbean people living in south London.

On the other hand, other studies support that GDS-SF results are not equivalent in different cultures, since the concept of depression and the forms of expression of positive and negative emotions may vary in each culture [65]. The same may occur with concepts such as “worthlessness” and “hopelessness” [66]. Moreover, the clinical presentation of depression and anxiety is also culturally variable [67]. Jang Y. et al. [68] argue that cultural differences may affect the participants understanding of the GDS-SF questions, highlighting, for example, that a positive response to the question “Do you prefer to stay at home, rather than going out and doing new things?” does not necessarily indicate a depressive symptom as it does for an older adult in the United States. This may be the case for Munduruku people as well.

We did not establish a cutoff score to diagnose depression in this study because the Munduruku have sociocultural specificities and the GDS-SF was applied in a predominantly young population. The GDS-SF was used as a research screening tool and not as a diagnostic tool. Therefore, the scores obtained in the scale can only provide partial indicators of the participants’ mental health status. In other words, the scores obtained on the scale can only provide “clues” about the mental health conditions and Good-Living in the communities; it is not possible to establish any diagnosis or conclusive statement about it.

To our knowledge, there are no quantitative instruments to assess mental health indicators that were validated for Amerindian populations. This may be due to the scarcity of research on this subject but also to the fact that Amerindian people have medical systems and concepts of personhood, health, and territory that differ from those postulated in biomedical or psychological sciences. Additionally, in biomedical or psychological sciences, there is no consensus on concepts and research instruments to assess “well-being”, “happiness”, or “sadness”, for example [69].

From a clinical point of view, the conversations held with the participants during fieldwork provided exceptional data. However, there was no time available to conduct such conversations or non-structured interviews in a systematic way in order to produce robust qualitative data. Hereupon, qualitative research based on ethnographic and participative methodology may be interesting to investigate the relationship between methylmercury exposure and “mental health” indicators in Amerindian communities. Articulating quantitative and qualitative research may produce more substantial data. For example, based on this experience, it could be interesting to build a semi-structured questionnaire together with people from the Munduruku communities. It could also be interesting if, in addition to participating in the elaboration of the questionnaires, Munduruku people were the ones to apply it.

### 4.4. Impacts of Goldmining on Munduruku People Good-Living (Xipan Jewewekukap)

As was discussed above, the concepts of “well-being”, “happiness”, and “sadness” vary greatly between different cultures, as well as the symbolic forms used to represent it. For the Munduruku people, what biomedical and psychological sciences define as “mental health” is deeply intertwined with the maintenance of a Good-Living. As such, to understand how goldmining is affecting Munduruku people beyond the impacts of methylmercury contamination, it is important to understand what makes a Good-Living—a *Xipan Jewewekukap*—according to the Munduruku people that live in the villages where the research took place.

Elders and leaders that were interviewed talked about the complexity of the problems caused by mining. A village leader explained to the team that the ecological problems caused by goldmining are not limited to factors that non-Amerindian people tend to consider but are also related to cosmopolitical dynamics [70]:

Pajés *[traditional healers] learned what they know from snakes. They learned that human diseases come from antiquity, from the first times of the world—times when abysses were open. Many other diseases remained at the bottom of the earth when these abysses closed. Nowadays, since* pariwat *(non-Amerindian people) are rummaging deep in the earth, these diseases are rising. If* pariwat *continue to rummage where they shouldn’t, all of us, Munduruku and* pariwat*, will have serious problems.*

The goldmining activities, as well as logging, grabbing, and the construction of hydroelectric dams, have been destroying places that are sacred for the Munduruku [71]. Leaders and elders report that some of the affected places are the homes of powerful entities, which are becoming angry at the destruction of their dwelling-place. Enraged with such disrespect, those entities can cause many diseases and deaths among the Munduruku. Such cosmopolitical imbalance caused by goldmining activities at the Munduruku territory is intertwined with the phenomena of methylmercury contamination. A *pajé* that works and lives in one of the communities visited explained:

*I am a* pajé*, but I don’t know how to cure problems caused by mercury contamination. This is new to me. Nevertheless, I know how to take care of illnesses that are caused by the fact that* pariwat *are messing with places they shouldn’t. They are messing with other beings’ houses and those beings are getting very angry. That is why we need to combine Munduruku medicines with* pariwat *medicines. And that is why we have to combine efforts to stop* pariwat *from messing with the other being’s houses.*

In addition to damaging the relationships that Munduruku people carefully maintain with other forest-dwelling beings, the goldmining activities affect structural aspects of the Munduruku cosmos. A Munduruku Health Agent of the *Poxo Muybu* and *Sawré Muybu* communities told the team stories about the beginning of the world. He explained:

*Everything in this world has a mother: the fish, the water, the stone... When we mess with the mothers’ offspring, they get angry and attack us, making us ill. Making these mothers mad is very dangerous for the Munduruku. Unfortunately, the destruction of the forest brings even more serious problems than those illnesses. I have learned from the elders that, since the beginning of the world, there is an enormous tree that supports the sky, preventing it from falling to the earth. The miners and loggers are cutting the nails that are at the roots of this tree. If that tree falls, the sky will fall and this world we live in will end.*

In this regard, this Munduruku Health Agent emphasizes that fighting against the destruction of the forest is a matter of life or death not only for the Munduruku people but for non-Amerindian people as well. Consequently, it is urgent to combine efforts to prevent an ecological collapse or the fall of the sky [72,73,74]. The invasion of Munduruku territory by non-Amerindian people brought several problems that did not exist before. Since those problems are increasing at great speed, the Munduruku people are calling on *pariwat* to take responsibility and to collaborate in facing those problems.

## 5. Conclusions

This research team has made the effort to examine the impacts of illegal goldmining on Amerindian villages’ Good-Living and explore the possible relationship between methylmercury exposure and the worsening mental health conditions in areas under the long-lasting threat of invaders.

Our data point to a tendency in which higher levels of methylmercury exposure could be linked to worse mental health indicators. Nevertheless, the GDS-SF does not seem to be a reliable data collection instrument regarding the Munduruku sociocultural context. However, qualitative research indicates an evident relationship between the impacts of illegal goldmining, Munduruku people’s worsening mental health conditions, and an increasing difficulty in maintaining a self-determined Good-Living. Such results, thus, point to the importance of carrying out new investigations, especially considering longitudinal studies with qualitative methodologies articulated to ethnographic research.

## Figures and Tables

**Table 1:** Sociodemographic and clinical variables of the study participants, according to GDS-SF. *Sawré Muybu* Indigenous Land, Pará, Brazilian Amazon, 2019.

	GDS-SF						
	<4		≥4		Total		
**Sociodemographic Features**							
Sex	*n*	%	*n*	%	*n*	%	*p*-value
Male	39	69.6	17	30.4	56	51.4	0.416
Female	33	62.3	20	37.7	53	48.6	
Total	72		37		109		
Age range							
≥30 years	23	67.6	11	32.4	34	31.2	0.376
20 to 29 years	22	57.9	16	42.1	38	34.9	
12 to 19 years	27	73.0	10	27.0	37	33.9	
Total	72		37		109		
Schooling							
>9 years	17	68.0	8	32.0	25	22.9	0.339
5 to 9 years	47	69.1	21	30.9	68	62.4	
0 to 4 years	8	50.0	8	50.0	16	14.7	
Total	72		37		109		
Marital Status							
Married	49	64.5	27	35.5	76	69.7	0.597
Single/Widow	23	69.7	10	30.3	33	30.3	
Total	72		37		109		
Villages							
*Sawré Muybu*	24	52.2	22	47.8	46	42.2	0.002
*Poxo Muybu*	34	87.2	5	12.8	39	35.8	
*Sawré Aboy*	14	58.3	10	41.7	24	22.0	
Total	72		37		109		
Regular Income							
Yes	27	67.5	13	32.5	40	36.7	0.808
No	45	65.2	24	34.8	69	63.3	
Total	72		37		109		
**Clinical Features**							
Nervousness	n	%	n	%	n	%	*p*-value
No	61	74.4	21	25.6	82	75.2	0.001
Yes	11	40.7	16	59.3	27	24.8	
Total	72		37		109		
School failure							
No	41	74.5	14	25.5	55	51.9	0.055
Yes	29	56.9	22	43.1	51	48.1	
Total	70		36		106		
Irritability							
No	69	71.9	27	28.1	96	88.1	0.001
Yes	3	23.1	10	76.9	13	11.9	
Total	72		37		109		
Physical Activity restriction							
No	68	68.0	32	32.0	100	91.7	0.153
Yes	4	44.4	5	55.6	9	8.3	
Total	72		37		109		
Previous Malaria							
No	38	73.1	14	26.9	52	47.7	0.139
Yes	34	59.6	23	40.4	57	52.3	
Total	72		37		109		
Previous hospitalization							
No	44	68.8	20	31.3	64	58.7	0.479
Yes	28	62.2	17	37.8	45	41.3	
Total	72		37		109		

**Table 2:** Clinical and sociodemographic variables, according to GDS-SF scores ≥ 4 (Crude Poisson Regression Model). *Sawré Muybu* Indigenous Land, Pará, Amazon, Brazil, 2019.

GDS-SF ≥ 4		
Variables	PR Crude (CI 90%)	*p*-Value
**Hg Level**		
Hg < 10 µg/g	1.0	
Hg ≥ 10 µg/g	1.6 (1.0–2.5)	0.072
**Nervousness**		
No	1.0	
Yes	2.3 (1.5–3.5)	0.001
**Irritability**		
No	1.0	
Yes	2.7 (1.9–3.9)	0.001
**Regular Income**		
Yes	1.0	
No	1.1 (0.7–1.7)	0.809
**Villages**		
*Sawré Muybu*	1.0	
*Poxo Muybu*	0.3 (0.1–0.6)	0.003
*Sawré Aboy*	0.9 (0.5–1.4)	0.630
**Age range**		
≥30 years	1.0	
20 to 29 years	1.3 (0.8–2.2)	0.399
12 to 19 years	0.8 (0.5–1.5)	0.624
**Gender**		
Male	1.0	
Female	1.2 (0.8–1.9)	0.418
**Schooling**		
>9 years	1.0	
5 to 9 years	1.0 (0.6–1.7)	0.918
0 to 4 years	1.6 (0.8–2.9)	0.245
**School Failure**		
No	1.0	
Yes	1.7 (1.1–2.7)	0.061
**Marital Status**		
Married	1.0	
Single/Widow	0.9 (0.5–1.4)	0.603

**Table 3:** Clinical and sociodemographic variables, according to GDS-SF scores ≥ 4 (adjusted Poisson regression model). Sawré Muybu Indigenous Land, Pará, Amazon, Brazil, 2019.

GDS-SF ≥ 4		
Variables	PR-Adjusted (CI 95%)	*p*-Value
**Hg Level**		
Hg < 10 µg/g	1.0	
Hg ≥ 10 µg/g	1.8 (1.1–3.0)	0.024
**Nervousness**		
No	1.0	
Yes	2.1 (1.3–3.3)	0.003
**Irritability**		
No	1.0	
Yes	3.0 (1.9–4.9)	0.001
**Regular Income**		
Yes	1.0	
No	1.1 (0.6–1.9)	0.766
**Age range**		
≥30 years	1.0	
20 to 29 years	1.4 (0.8–2.5)	0.219
12 to 19 years	0.7 (0.4–1.5)	0.400

## Data Availability

Data sharing not applicable.
